# Equitable partnership: patient and public involvement and engagement and community engagement in mental health research in Pakistan

**DOI:** 10.1186/s40900-026-00893-6

**Published:** 2026-06-19

**Authors:** Shumaila Hamid, Syeda Fatima Jamal, Syed Muhammad Uzair Shah, Ishfaq Tariq Azeemi, Abdul Jalil Khan, Saima Sheikh, Taha Ayub, Nishani Fonseka, Abbie Milner, Mian Mukhtar Ul Haq Azeemi, Muhammad Firaz Khan, Sherdil Khan, Ahmad Ali Nauman, Noor Sanauddin, Saeed Farooq, Krysia Canvin

**Affiliations:** 1 Institute of Public Health and Social Sciences, Khyber Medical University, Peshawar, Pakistan; 2 Institute of Family Medicine, Khyber Medical University, Peshawar, Pakistan; 3 Nuffield Department of Orthopaedics, University of Oxford, Oxford, UK; 4 School of Medicine, Keele University, Staffordshire, UK; 5 Medical Teaching Institute, Lady Reading Hospital, Peshawar, Pakistan; 6 Institute of Public Mental Health and Behavioral Sciences, Khyber Medical University, Peshawar, Pakistan; 7 Medical Teaching Institute, Khyber Teaching Hospital, Peshawar, Pakistan; 8 Prime Institute of Public Health, Peshawar, Pakistan; 9 Department of Sociology, University of Peshawar, Peshawar, Pakistan; 10 Research Fellow in Involvement & Engagement, School of Medicine, Keele University, Staffordshire, ST5 5BG UK; 11 Institute of Family Medicine, Khyber Medical University, Peshawar, Pakistan

**Keywords:** PPIE, Community engagement, Psychosis, Early intervention, Mental health research, LMICs, Traditional healers, Primary care

## Abstract

**Background & aim:**

Patient and public involvement and engagement (PPIE), alongside community engagement, are crucial in mental health research to ensure relevance and impact. This study, part of the Medical Research Council (MRC) UK-funded, *T*raditional *HE*alers working with primary care and mental *H*ealth for early intervention in *P*sychosis in young p*E*rsons (THE HOPE) project, describes the implementation of PPIE and community engagement in THE HOPE project and their impact on the project and community.

**Methods:**

A six members advisory group, including individuals with lived experience of psychosis, caregivers, and a TSH, contributed to the study design, topic guides, and community engagement initiatives. Thirty-four community engagement sessions were conducted in four phases, utilizing sermons, posters, pamphlets, and direct outreach.

**Results & conclusion:**

The thematic analysis highlighted key issues like medication adherence, service delivery, and the role of traditional healers and primary care. Insights from the advisory group informed recommendations for shared decision-making and capacity-building, emphasizing the importance of inclusive, culturally relevant, and collaborative approaches in mental health research.

**Supplementary information:**

The online version contains supplementary material available at 10.1186/s40900-026-00893-6.

## Abstract (plain language summary)

People living with psychosis often experience stigma and discrimination, which act as barriers to accessing appropriate health care needs. As part of THE HOPE Feasibility Study, we aimed to strengthen partnerships between researchers, patients, carers, and the general community in Peshawar, ensuring that individuals with lived experience of psychosis actively contribute to the research process from inception to implementation. A Patient and Public Involvement and Engagement (PPIE) group, consisting of six members, including individuals with lived experience of psychosis, caregivers, and a TSH, the Lived Experience Advisory Panel (LEAP), was established to provide regular input on study intervention development and the community awareness sessions. Capacity-building sessions were organized for both researchers and advisory members to understand the importance of patient and public involvement in research. Additionally, community engagement sessions were conducted to explore perceptions of psychosis, experiences, myths, stigma, and barriers to accessing health care. Feedback from the advisory group and community engagement sessions helped in the development of culturally sensitive intervention and awareness campaigns. THE HOPE Study’s PPIE and CEI initiatives demonstrate how meaningful public partnerships can lead to more relevant and effective mental health research in low-resource settings.

**Supplementary information:**

The online version contains supplementary material available at 10.1186/s40900-026-00893-6.

## Introduction

Patient and public involvement and engagement (PPIE) in research can be defined as research carried out “with” or “by” members of the public rather than “to”, “about” or “for” them [1]. while Community Engagement and Involvement (CEI) refer to the active involvement of communities throughout the research process, utilizing participatory approaches and collaborating with key stakeholders to enhance the relevance and conduct of health research [2].

Effective research is characterized by active and meaningful public and patient involvement throughout the research process, ensuring that studies address the real-world needs and priorities of people with lived experience. This is important from both an ethical and practical standpoint, as it improves communication and makes research more accessible, acceptable, and relevant, thereby increasing uptake of findings. Implementing public involvement requires considerable effort; hence, early planning and resources are crucial for stakeholders to contribute meaningfully [3, 4].

PPIE is essential in mental health research because it ensures that studies address the real-world needs and priorities of people with lived experience, thereby increasing relevance and uptake of their findings [5]. Co-production of interventions can help overcome known challenges in Severe Mental Illnesses like psychosis, such as low motivation and reduced physical activity, subsequently improving acceptability or even effectiveness [6]. Systematic reviews suggest that service user involvement in mental health services and delivery can lead to measurable improvements in the quality and effectiveness of care [7]. PPIE is gaining increasing importance in psychiatric research; however, research and practice in this area remain at an early stage in some fields and in LMICs [8]. CEI complements PPIE by bringing together diverse stakeholder groups, and engaging community members throughout the process enables open communication, promotes trust, helps address cultural barriers and stigma, and ensures research meets community needs. Integrating PPIE and CEI strategies has the potential to make mental health research more ethical, impactful, and culturally sensitive [9].

In many High-Income Countries (HICs), PPIE is increasingly practiced in research, whereas it remains a relatively new idea that is seldom utilized in LMICs [10]. There is a lack of reporting on PPI strategies and impact, as studies often present only the researcher’s perspective, resulting in PPIE being frequently underreported or absent [11]. Despite the potential for CEI to transform mental health systems in LMICs, Western models often require adaptation to align with local cultures and values for successful implementation, and guidance on community and public engagement should therefore respect cultures and adapt research to local contexts. Global health research is still largely led by academics in HICs, where social, cultural, and economic contexts differ from those in LMICs. Involving diverse local actors from patients to policymakers is essential for bridging the gap between medical knowledge and practical application in health systems in various LMICs [12].

In THE HOPE project, PPIE was operationalized through the Lived Experience Advisory Panel (LEAP), while CEI referred to the application of LEAP’s insights and recommendations through community engagement activities and public-facing initiatives. THE HOPE Research project is a novel intervention program aimed at testing a culturally appropriate intervention for early identification, referral, and management of First Episode Psychosis in the young population, and to evaluate its feasibility for implementation in Peshawar, Khyber Pakhtunkhwa, Pakistan [13]. This project involved Traditional and Spiritual Healers (TSHs) working collaboratively with Primary Care Physicians (PCPs) and psychiatrists through task-shifting for early detection, referral, and treatment of First Episode Psychosis [13]. THE HOPE project actively involved the PPIE group, titled the LEAP, which provided feedback on the study design, feasibility trial phase, and process evaluation, and suggested planning and conducting CEI to raise awareness regarding psychosis, promote early care seeking, and reduce stigma. PPIE and CEI were particularly necessary in this project to ensure community understanding, build trust, and adapt the intervention to the cultural context.

This paper describes the implementation of PPIE and community engagement in THE HOPE project and their impact on the project and community.

## Methods

### Study design

Our study employed a participatory approach combining PPIE and CEI.

### Approach to PPIE in the HOPE project

We identified and recruited a diverse PPIE group considering the target population over a period of three months through professional contacts, public engagement events, and by directly approaching trial participants, patients, and families attending our study. PPIE members were approached via phone and personal meetings to request their support. Commonly cited strategies for recruiting PPIE members via patient-led committees, charities, or advocacy groups were not possible, as no such groups exist locally.

While our project team did not have any strict eligibility criteria for choosing the PPIE members, we strived for diversity in terms of sex, ethnicity, educational background, and attitudes towards research, and assessed whether potential members had motivation, interest, and confidence to voice opinions. These characteristics have been previously identified as crucial indicators of PPIE members’ ability to effectively participate in discussions, co-lead the group, and sustain their involvement.

We also felt strongly that it was our responsibility to make PPIE in research accessible to all and reduce bias inherent among members in the PPIE group. Potential members had an informal discussion with the Co-PI to understand motivations for joining the PPIE group and confirm that individuals could commit the time required to attend meetings every 4–6 weeks. In the absence of an established consensus on the optimal group size for Patient and Public Involvement and Engagement (PPIE) in research, a group of five to seven members was considered appropriate. This size was selected to ensure inclusion of diverse perspectives while maintaining a manageable and supportive environment conducive to meaningful engagement and the development of effective working relationships.

The PPIE group for our study consisted of six members from various backgrounds who had experienced mental health conditions and or were in a caring capacity for family members with mental health conditions. The group had four males and two females, including individuals with lived experience of psychosis, caregivers, and a TSH. Hence, the PPIE group was renamed THE HOPE Lived Experience Advisory Panel (LEAP).

All LEAP meetings were scheduled at Khyber Medical University (KMU), Pakistan, an easily accessible venue agreed upon by all members. This facilitated convenient attendance for LEAP participants. An initial LEAP meeting was scheduled to introduce the members to each other and the research team. This meeting explained and clarified the participation of a LEAP member as distinct from being a study participant and focused on providing in-depth but jargon-free information about THE HOPE research project. LEAP members were given enough time (at least a week for an hour’s work) to review and provide feedback on other documents. LEAP provided feedback through consensus discussions and iterative reviews. Feedback was provided in local languages (Pushto, Urdu). To ensure accessibility and inclusivity, flexible timing and dates were offered for LEAP meetings to accommodate the needs of the group. Regular communication was maintained with the group through newsletters, regular meetings, and updates on activities, planned activities, and timelines. Feedback was collected through face-to-face meetings, telephone interviews, and email correspondence to accommodate individual preferences. Both group and one-to-one meetings were conducted to gather their feedback. The GRIPP2 Checklist (Guidance for Reporting Involvement of Patients and the Public 2) was used for reporting of the PPIE in our study.

### First Session: The HOPE lived experience advisory panel (LEAP)

The first session aimed to connect with the diverse LEAP group of individuals with lived experience of psychosis, caregivers, and a TSH, all with a shared interest in improving the lives of those living with psychosis. We opted for group discussion with an agenda for open dialogue with an emphasis on confidentiality and respect for their experiences and contributions. Data was collected through session attendance records and observational notes. Our goal for the discussion was twofold: firstly, to promote interaction among the LEAP members and THE HOPE research and field team to enhance collaboration between various stakeholders in the psychosis care landscape, and secondly, to discuss the role of the LEAP group, expectations, and what their involvement would be throughout the project. They were also briefed about community engagement initiatives, their importance in research, and the activities undertaken by THE HOPE team regarding psychosis in Peshawar. To facilitate engagement, the session incorporated experience sharing followed by a question-and-answer session.

### Second session: refining topic guides for process evaluation through co-production: a cornerstone of patient and public involvement in the HOPE research project

The second session aimed to solicit feedback and input on the formulated topic guides for the process evaluation of the project through the collaborative approach of co-production, where researchers, patients, and professionals sit together to discuss research and service design. The 2-hour discussion was divided into two halves. The first half focused on establishing a shared understanding of the project’s needs and impacts related to process evaluation. By placing the LEAP members at the forefront of decision-making, co-production aimed to foster inclusivity, shared power, and mutual respect. This approach utilized the value of diverse knowledge and expertise, bridging the gap between research and real-world needs. The second half focused on revising and refining the topic guides. For psychiatrists and primary care physicians’ topic guides, a comprehensive review of each question was conducted.

The feedback was collected through open discussion and note-taking. Collective input from LEAP informed the research design and outcomes while valuing diverse perspectives and expertise. In THE HOPE research project, co-production was integral to the LEAP. Our project embodied co-production principles where patients, caregivers, and community stakeholders collaborated with researchers and professionals.

### Third Session: Community engagement and awareness strategy development through lived experience advisory panel (LEAP)

**Fig. 1:**
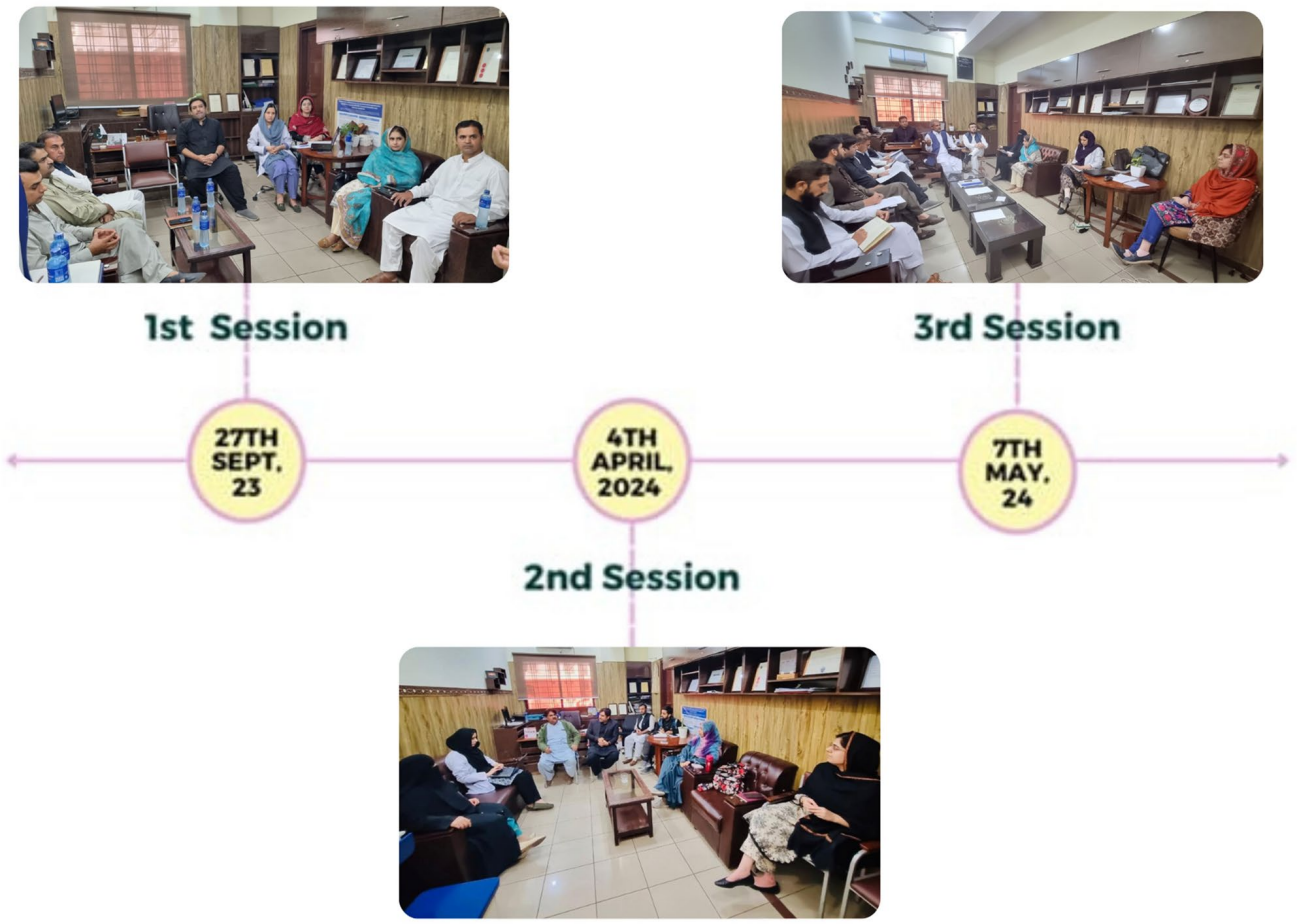
Shows three sessions of the research team with the HOPE lived experience advisory panel LEAP

The third LEAP session commenced with updates on the trial and process evaluation. Building on the previous session’s outcomes, the revised topic guides incorporating the suggestions by LEAP members were presented. They shared their experiences participating in THE HOPE research project, providing valuable insights for enhancing the CEI’s aims and goals. THE HOPE team presented a comprehensive plan, integrating LEAP recommendations, to the Principal Investigator (PI) and LEAP members. To promote community awareness, culturally tailored awareness materials were shared with the members that included: (1) Sermon: A religious scholar crafted a sermon to be delivered by Imams, leveraging Islamic teachings to reach the masses (Annex 2), and Posters in Urdu were designed to convey project messages (Annex 1). A pictorial representation of the LEAP meeting sessions timeline is shown in Fig. 1.

### Study setting

CEI activities took place in the city of Peshawar, Khyber Pakhtunkhwa province, Pakistan. We utilized mosques as dissemination platforms for sermons (Khutbah) and distribution of informational pamphlets along with the community centers, traditional/spiritual healers’ spaces, Universities/colleges, and social media platforms.

### Approach to community engagement initiative: promoting psychosis awareness in Peshawar

We conducted a total of 34 community engagement (CE) sessions between October 2022 and March 2024, in the city of Peshawar, Khyber Pakhtunkhwa province, as part of a comprehensive community engagement initiative under THE HOPE research project. The community engagement sessions were conducted in study sites selected to ensure representation of the target population and relevance to the research objectives. The CEI was implemented in four distinct phases: (1) psychosis awareness in the general community, (2) engagement with traditional and spiritual healers, (3) youth mental health awareness in educational institutions, and (4) broader community-level mental health awareness. Phases were developed collaboratively with LEAP and the research team based on community needs and engagement goals. A description of the design, target population, and participants is given in Table 1.

**Table 1:** Outlines the four phases of the community engagement initiative, focusing on four key components: design, target population, participants, and community involvement. The table provides a breakdown of each component

Phase 1: Psychosis Awareness	Design	Target population	Participants	Community involvement
	Ten community engagement sessions across four Sub-districts	General community (six male-focused, four female-focused sessions)	90 individuals (52 males, 38 females)	Nazims (local community leaders)
*Phase 2: Engaging Traditional Healers*	Four sessions with traditional and spiritual healers across local government units.	Traditional and spiritual healers (TSHs) and the community	210 individuals	TSHs
*Phase 3: Youth Mental Health Awareness in Educational Institutions*	Three sessions in educational institutions	Youth (University of Peshawar, Shaheed Benazir Bhutto Women University, Government College of Technology Peshawar)	400 students (280 males, 120 females)	Head of educational institutions, Director of elementary and secondary education, Director of Higher Education
*Phase 4: Mental Health Awareness in the General Community*	17 community-level sessions in Hujras (traditional gathering spaces), mosques, and primary care facilities	General community (community elders, religious leaders, government officials)	1600 individuals	Imams utilized informational pamphlets to the congregation during Friday prayers

A multidisciplinary team of experts in psychiatry, primary care, public health, and sociology facilitated the sessions. The main objective was to raise awareness about psychosis among local communities, emphasizing early identification, referral, and management of first-episode psychosis in adolescents. This initiative demonstrated successful community engagement and awareness sessions regarding psychosis. The involvement of local leaders and expert facilitators ensured contextual relevance and effectiveness. This community engagement initiative underscores the importance of targeted awareness campaigns in promoting mental well-being and reducing stigma. Sessions were aimed at fostering trust and collaboration with the TSHs community, leveraging their influence to promote awareness, identification, detection, and early referral of First-Episode Psychosis (FEP) patients to tertiary care. By engaging TSHs, the project established vital connections with community influencers, enhanced awareness of FEP detection and referral, and encouraged collaboration between traditional and standard healthcare providers. This initiative demonstrates the feasibility and importance of involving traditional healers in mental health care. By building bridges between standard and traditional healthcare systems, we can improve early detection, treatment, and outcomes for individuals experiencing First Episode Psychosis. On World Mental Health Day, THE HOPE research project’s Chief Investigator appeared on a national TV show, emphasizing the importance of mental health awareness, particularly among youth, and urging authorities and communities to address this critical issue. THE HOPE project’s community engagement initiative engaged 2,300 participants across 20 venues, building collaborations with TSHs, educational institutions, and community leaders, and promoting mental health awareness and early intervention.

### Data analysis

For analysis, observational notes and feedback were analyzed thematically. Reflexivity was managed through regular team discussions and acknowledging the researcher’s positions.

## Results

### Learnings from leap

The LEAP sessions yielded rich insights from individuals with lived experience, caregivers, and stakeholders. Key insights included identifying challenges faced by people living with psychosis and caregivers, the importance of community engagement and involvement in psychosis care, medication adherence, service delivery, and primary care’s role in diagnosis, accessing services, and multidisciplinary teamwork involving TSHs, PCPs, psychologists, and psychiatrists, and improving health outcomes through collaborative care.

Members took an active part in the discussion and suggested conducting and expanding community engagement sessions regarding mental health across Peshawar. Two of the LEAP advisory members showed interest in participating with the research team in CEI activities. LEAP members provided feedback on technical documents such as topic guides (Annex 3–5), which also provided them with additional information and support to enable them to understand more technical content.

Members suggested incorporating a question regarding ethical concerns or dilemmas encountered by healthcare professionals during collaboration with TSHs, i.e., *(Can you describe any challenges or conflicts you’ve faced when working with TSHs*,* and how you’ve navigated them? )*. They recommended removing the question of the alignment of the project with religious beliefs from the Traditional and TSHs topic guide, deeming it unnecessary as it is not directly relevant to the project’s objectives and might make participants uncomfortable discussing personal beliefs, which could impact the quality of the discussion. Additionally, they suggested rephrasing a question concerning opportunity costs. The advisory members endorsed the questions but advised simplifying and elaborating upon them for enhanced comprehension by patients and carers.

LEAP reviewed these materials, suggesting contextual modifications to enhance local understanding, avoiding stigmatizing language related to mental health, using culturally sensitive language, and emphasizing awareness without perpetuating negative stereotypes. The session resulted in a consensus on utilizing Khutbah (Sermon) as a primary awareness strategy, supplemented by posters. THE HOPE team refined the materials, incorporating LEAP feedback. They have been actively involved in the dissemination of study findings (posting on patient forums and participating in media interviews). They reviewed and provided feedback on the study findings.

### Insights and impact from the LEAP

#### Power dynamics and challenges

Our project implemented strategies to promote equal partnerships through co-production approaches, ensuring shared decision-making, training, and capacity-building programs supported by public and patient contributors, flexible engagement methods accommodating diverse needs, and regular feedback resulting in open communication. By acknowledging and addressing power dynamics, THE HOPE project demonstrates the feasibility of meaningful LEAP in mental health research.

#### Collaborative solutions and community awareness

The LEAP sessions facilitated open dialogue, generating innovative solutions such as increasing community awareness through masjid imams’ sermons, reaching undiagnosed cases through targeted outreach, enhancing research relevance through patient-centered perspectives, fostering collaboration between researchers, caregivers, TSHs, and people with psychosis, and ultimately bridging the gap between research and real-world psychosis care.

#### Enhancing community engagement and reach

LEAP advisory members emphasized the importance of expanding community engagement and involvement (CEI) to promote mental health awareness effectively. Key suggestions included targeting larger populations through media, social media, and CEI sessions in schools and universities, involving Imams and Nazims to leverage their influence and credibility, regular mental health awareness programs to ensure sustained impact, building a network of people to continue the awareness process, and targeting female populations, particularly female students in Madrasas, to address limited access to information. THE HOPE research team conducted multiple CEI sessions in educational institutions and local communities in line with the strategic outreach plan advised by the LEAP, creating partnerships with locals and increasing the impact of the sessions.

#### Cultural adaptation and accessibility

LEAP members emphasized the importance of cultural sensitivity and accessibility in CEI approaches and materials. The research team addressed all the recommendations by the LEAP members. Topic guides were translated into local languages (Pushto, Urdu) to overcome language barriers, incorporated cultural relevance to destigmatize it to resonate with psychosis patients and carers, simplified medical terminologies into layman’s terms for better understanding, and addressed the specific needs of the female population with limited access to information by conducting CEI sessions with female students. This theme underscores the importance of adapting CEI initiatives to the local linguistic context to ensure gender-balanced reach.

### Learnings from CEI

Community Engagement and Involvement (CEI) Phase 1 insights reflect the need for sustainable mental health solutions that are accessible to all. The community members proposed different key strategies to support individuals from disadvantaged backgrounds living with psychosis and other mental health disorders. The strategies were to expand community engagement activities across different cities and establish free mental health consultation services along with medicine supplies for those in need. They also suggested developing advocacy and social support groups with the help of local and provincial leaders addressing severe mental disorders, using social media and mobile for widespread mental health awareness messages, introducing mental health screening in schools and colleges, launching awareness campaigns to reduce the stigma associated with mental health issues, empowering community representatives such as community elders, masjid imams, and local government representatives to disseminate mental health awareness within their networks. These community-driven solutions align with THE HOPE project’s goals, emphasizing the importance of community-centered approaches through CEI, sustainable mental health services, digital innovation, and stigma reduction.

#### Collaborative community engagement through traditional and spiritual healers (TSHs)

In CEI Phase 2, TSHs participated and collaborated with THE HOPE research team on mental health improvement initiatives. TSHs played an integral role in promoting psychosis awareness within their communities. Pamphlets on psychosis and mental health were distributed during mosque-based CEI activities, along with posters in local languages (Pushto, Urdu), which were placed in primary care facilities such as Basic Health Units and Local government units based on TSHs’ recommendations. These approaches suggest the potential for faith-based interventions and community-led initiatives to address mental health stigma and promote awareness. This TSH-led initiative aligns with THE HOPE project’s objectives, highlighting the importance of community-TSH partnerships, culturally sensitive awareness materials, and faith-based interventions. By integrating traditional and spiritually driven approaches, THE HOPE project developed context-sensitive materials addressing psychosis and mental health issues in local communities.

#### Community-driven solutions for sustainable mental health care

In CEI Phase 3, students emphasized the need for continuous support for individuals with mental health disorders. They proposed targeted awareness initiatives focusing on common mental health issues and treatment options, particularly among youth. Key recommendations that were adopted by THE HOPE research project included involving universities and colleges in awareness campaigns to reduce discrimination and stigma attached to mentally ill individuals, empowering communities, especially youth, with relevant knowledge of mental health problems and treatment options, and expanding the reach of mental health education to minimize social exclusion.

#### Building trust and community awareness through collaborative mental health education

The CEI Phase 4 insights underscored the effectiveness of engaging religious leaders in community education sessions, fostering trust between researchers and community participants. Key outcomes of this phase were recognition of mental health issues and their negative impacts on individuals and communities, increased understanding of the interconnection between mental and physical well-being, and emphasis on early intervention of psychosis for a healthy, fulfilling life.

## Discussion

The phrase “Mind the Gap” serves as a reminder of the disconnect between policy and practice in patient and public involvement and engagement in healthcare. THE HOPE research project addressed this gap by integrating a meaningful Lived Experience Advisory Panel (LEAP) into its mental health research, demonstrating that both patients and communities can be actively involved in research and intervention design, as the outcomes ultimately affect them. The sessions yielded valuable insights into real-world challenges faced by people with psychosis and caregivers, highlighting the importance of community awareness and involvement in psychosis. This stakeholder feedback refined the topic guides, ensuring relevance and effectiveness in capturing insights for process evaluation, consistent with evidence that PPIE improves research design, relevance, and collaboration in health research. These findings reflect participatory approaches that emphasize active involvement of stakeholders in shaping research processes and outcomes. The findings of THE HOPE’s PPIE and CEI in mental health research emphasize the importance of integrating lived experience into research processes, as demonstrated through the LEAP, which helped bridge the gap between research and community needs by ensuring that the voices of patients, caregivers, and community stakeholders were central. This emphasis on co-production, service user leadership, and collaboration between service users, practitioners, and academics aligns with existing literature (Alison et al., 2019) [14], while also reinforcing findings from Thornicroft et al. (2022) [15] and Clark et al. (2023) [16], which highlight the importance of incorporating lived experience to reduce stigma, improve access to care, and influence decision-making processes.

One of THE HOPE project’s key strengths was its commitment to addressing power dynamics through shared decision-making and engagement strategies, demonstrating how participatory approaches can ensure that stakeholder involvement is meaningful rather than symbolic. This reflects co-production approaches that emphasize power-sharing and meaningful involvement of stakeholders in research processes. This approach is consistent with Bell et al. (2021) [17], who emphasize that successful co-production in mental health research requires power-sharing and strategic planning to ensure genuine influence on outcomes.

THE HOPE project’s emphasis on cultural adaptation and accessibility represents an important contribution to mental health research in LMICs. Similar to findings from Irmansyah et al. (2020) [18], where cultural paternalism, stigma, and resource constraints limited implementation despite high awareness, this study demonstrates how adapting engagement strategies to local contexts is necessary for effective implementation. The approach also aligns with Wolf Linder et al. (2024) [19], emphasizing that effective dissemination requires active participation from PPIE members and community-driven knowledge translation strategies. By engaging LEAP members in shaping community engagement initiatives and dissemination strategies, and by translating materials into local languages, simplifying medical terminology, and addressing the needs of marginalized groups, the project ensured that its CEI initiatives were culturally and contextually relevant.

The community-driven solutions proposed by LEAP members, including expanding mental health awareness through media and social platforms, strengthening youth engagement, and developing long-term support services, reflect broader trends in LMIC mental health research emphasizing community-based and sustainable interventions. These findings collectively highlight the importance of community engagement, youth involvement, and culturally relevant approaches in strengthening mental health systems in LMICs. Studies such as Jumbe et al. (2022) [20], Duara et al. (2022) [21], and Wai et al. (2024) [22] similarly highlight the importance of youth engagement, culturally relevant awareness strategies, and involvement in decision-making processes. Additionally, evidence from Hall et al. (2020) [23] and Bhardwaj et al. (2020) [24] highlights the importance of involving families, service users, and community health workers in strengthening mental health systems and community engagement in low-resource settings. These findings are consistent with broader evidence from LMIC settings highlighting the role of participatory approaches and patient and public involvement in improving the relevance, quality, and implementation of health research. This is further supported by evidence demonstrating that community engagement is closely linked to political advocacy, social rights, and the shaping of public policy, while also promoting inclusive research practices that enhance the autonomy and empowerment of individuals living with mental illness (Florence et al. 2023) [25]. Additionally, systematic reviews in LMICs highlight that involving patients and caregivers can strengthen mental health systems (Semrau et al. 2016) [26].

## Limitations

THE HOPE project faced limitations, particularly in fully implementing CEI initiatives within the study’s timeframe. While most LEAP members’ recommendations were incorporated, some critical suggestions, such as engaging female madrasa (religious school) students and rural women, were not fulfilled. Given the significant barriers faced by women in the region regarding mental health literacy and service access, future efforts should prioritize targeted outreach to these populations.

Additionally, the LEAP consisted of a small group of six members, which may limit the representativeness of perspectives, as it may not fully capture the diversity of experiences within the wider community. There is also the potential for social desirability bias during community engagement activities, where participants may have provided responses that they perceived as acceptable or favorable, which could influence the findings.

Furthermore, sustainable CEI initiatives require structured long-term planning, engagement with local leaders, and continuous capacity-building efforts; however, these were beyond the scope and duration of the current study, raising questions about sustainability and scalability beyond the study period.

## Conclusion

THE HOPE project highlights the importance of integrating lived experiences and prioritizing cultural sensitivity in mental health research, particularly in psychosis care. Its findings reinforce the value of PPIE and CEI, offering practical strategies to make mental health care more inclusive and effective. By demonstrating the real-world impact of these approaches, the project provides a roadmap for advancing research and service design. Though there are limitations in the study, it sets a model for involving the community effectively in mental health research, and future studies should focus on sustaining these community-driven interventions, especially in low-resource settings.

## Electronic supplementary material

Below is the link to the electronic supplementary material.

Supplementary Material 1

Supplementary Material 2

## Data Availability

The data generated and analyzed during this study are not publicly available due to confidentiality and ethical considerations, but are available from the corresponding author on reasonable request.

## Abbreviations

LMIC: Low- and Middle-Income Country
PPIE: Patient and Public Involvement and Engagement
CEI: Community Engagement and Involvement
TSH: Traditional and Spiritual Healers
PCP: Primary Care Physicians
THE HOPE: Traditional HEalers working with primary care and mental Health for early intervention in psychosis in young pErsons
FEP: First Episode Psychosis
LEAP: Lived Experience Advisory Panel
MRC: Medical Research Council
KMU: Khyber Medical University
