# Supplementary material for: Equitable partnership: patient and public involvement and engagement and community engagement in mental health research in Pakistan
Source: Res Involv Engagem. 2026 Jun 19;12:97. doi: 10.1186/s40900-026-00893-6 (PMC13282870; doi:10.1186/s40900-026-00893-6)
Supplement: Supplementary file 1 — Supplementary Material 1 [file 40900_2026_893_MOESM1_ESM.docx]

**Supplementary Material: CEI SESSIONS TIMELINE**

The table below outlines the CEI sessions, including dates and locations.

| **S. No** | **CEI SESSIONS** | **Date** | **Location** |
| --- | --- | --- | --- |
| **1** | **1st Awareness session** | **2-Oct-2022** | **Bakshu Pull** |
| **2** | **2nd Awareness session** | **2-Oct-2022** | **Bakshu Pull** |
| **3** | **3rd Awareness session** | **5-Oct-2022** | **Muraqaba Hall** |
| **4** | **4th Awareness session** | **9-Oct-2022** | **Sufian Garden** |
| **5** | **5th Awareness session** | **9-Oct-2022** | **Sufian Garden** |
| **6** | **6th Awareness session** | **14-Oct-2022** | **Chamkani** |
| **7** | **7th Awareness session** | **14-Oct-2022** | **Chamkani** |
| **8** | **8th Awareness session** | **20-Oct-2022** | **Wahid Gari** |
| **9** | **9th Awareness session** | **26-Oct-2022** | **Tehkal** |
| **10** | **10th Awareness session** | **26-Oct-2022** | **Tehkal** |
| **11** | **World Mental Health Day Session** | **10-Oct-2023** | **University of Peshawar** |
| **12** | **Mental Health Awareness session** | **2-Nov-2023** | **Shaheed Benazir Bhutto Women University, Peshawar** |
| **13** | **1^st^ Awareness session** | **1-Dec-2023** | **Jamiya Masjid UC Lala** |
| **14** | **2^nd^ Awareness session** | **2-Dec-2023** | **Hujra, UC Ghari SherDad** |
| **15** | **3^rd^ Awareness session** | **3-Dec-2023** | **VC Nazim setting, Hassan Ghari** |
| **16** | **4^th^ Awareness session** | **4-Dec-2023** | **UC Mathra** |
| **17** | **5^th^ Awareness session** | **5-Dec-2023** | **UC Ander Shehr** |
| **18** | **6^th^ Awareness session** | **8-Dec-2023** | **Jamiya Masjid, UC Wadpaga** |
| **19** | **Meeting with the Director of Elementary & Secondary Education** | **11-Dec-2023** | **Directorate of Elementary & Secondary Education, Peshawar** |
| **20** | **7^th^ Awareness session with LHWs** | **12-Dec-2023** | **BHU, Lala kalay, UC lala** |
| **21** | **Session with youth** | **12-Dec-2023** | **Technical Institute, Warsak Road** |
| **22** | **8^th^ Awareness session** | **15-Dec-2023** | **Masjid/Madrasa Dheri Bhagbanan** |
| **23** | **Awareness session with LHWs** | **17^-^Dec-2023** | **BHU, UC Pishtakhara** |
| **24** | **Meeting with the Director of Higher Education** | **18-Dec-2023** | **Civil Secretariat, Peshawar** |
| **25** | **9^th^ Awareness Session** | **20-Dec-2023** | **Warsak Road** |
| **26** | **10^th^ Awareness Session** | **22-Dec-2023** | **Jamiya Masjid, Mathni** |
| **27** | **11^th^ Awareness Session** | **25-Dec-2023** | **VC Nazim Yakatooth** |
| **28** | **12^th^ Awareness Session** | **29-Dec-2023** | **Jamiya Masjid, Tarnab Farm** |
| **29** | **13^th^ Awareness Session** | **31-Dec-2023** | **VC Nazim Nahaqi** |
| **30** | **14^th^ awareness**  **Session** | **3^rd^ January 2024** | **Jamiya Masjid,**  **Rehan Abad, UC Landi Arbab** |
| **31** | **15^th^ awareness**  **Session** | **13^th^ January 2024** | **Afridiyano Ghari, UC Gulbahar** |
| **32** | **16^th^ Session arranged by Nazim** | **2^nd^ February 2024** | **UC Gulbela** |
| **33** | **17^th^ Awareness session** | **6^th^ February 2024** | **Governmental organization** |
| **34** | **18^th^ Awareness session** | **02^th^ March 2024** | **UC Mathra** |
