# Supplementary material for: Equitable partnership: patient and public involvement and engagement and community engagement in mental health research in Pakistan
Source: Res Involv Engagem. 2026 Jun 19;12:97. doi: 10.1186/s40900-026-00893-6 (PMC13282870; doi:10.1186/s40900-026-00893-6)
Supplement: Supplementary file 2 — Supplementary Material 2 [file 40900_2026_893_MOESM2_ESM.docx]

**Annexures:**

**Annex 1: Poster for CEI**
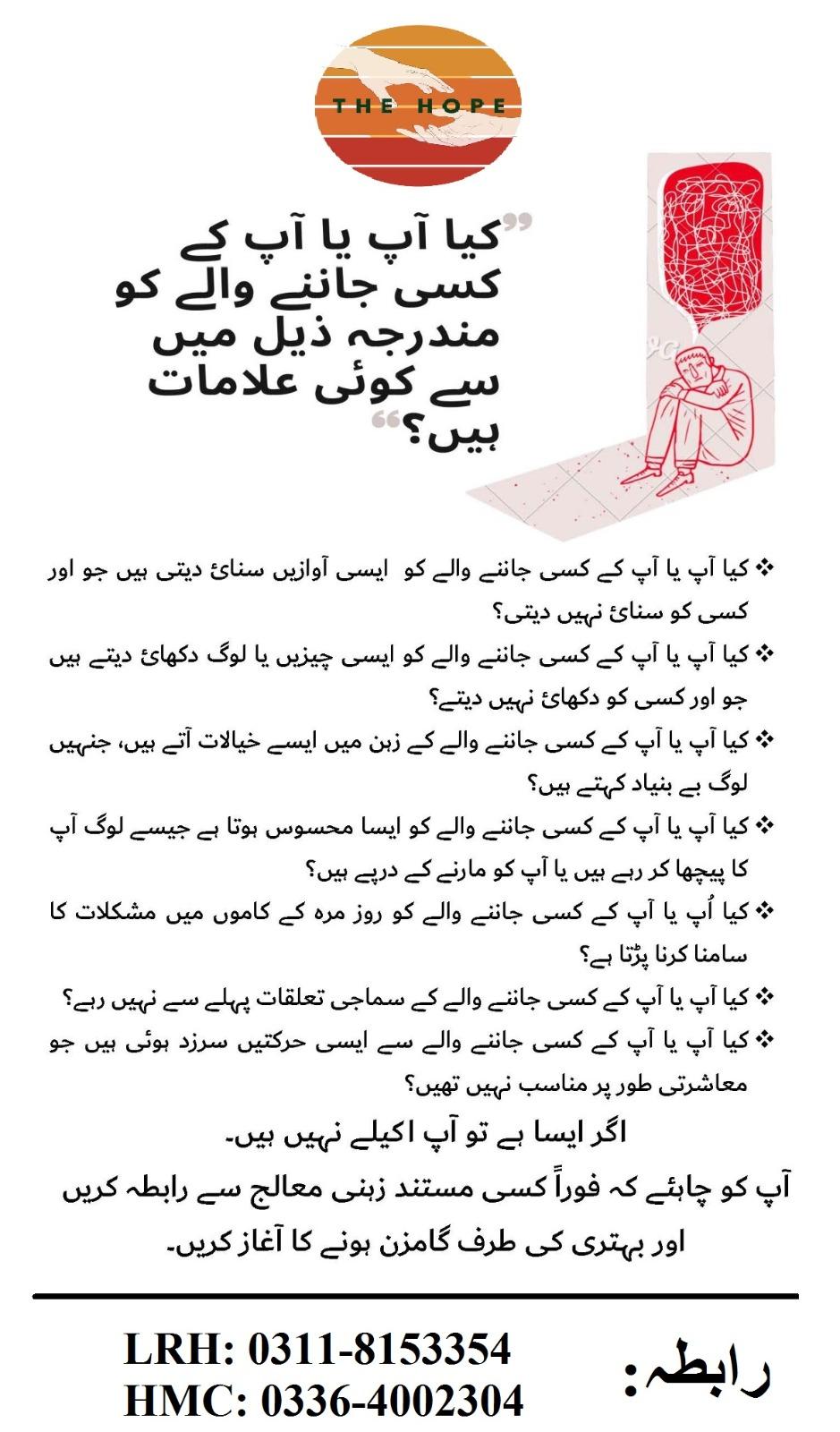
**(in Urdu and English)**

**
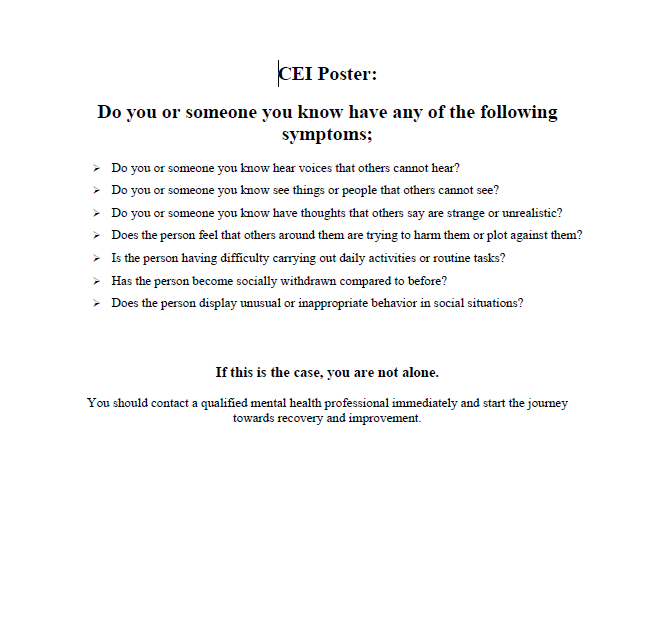
**

**Annex 2: Sermon (in Urdu and English)**


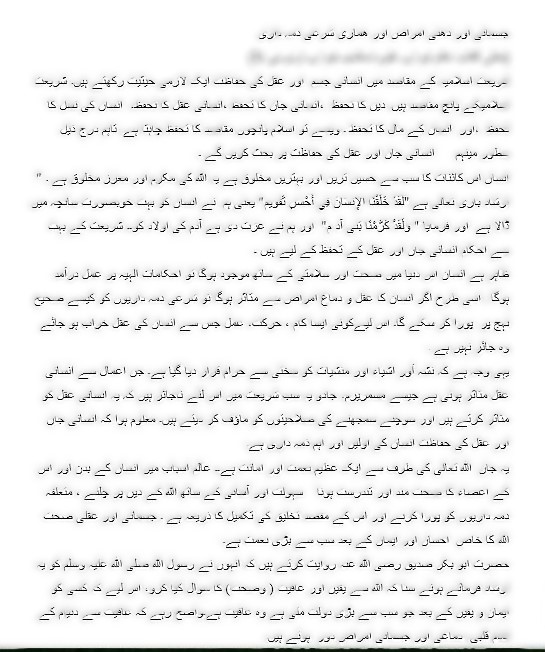


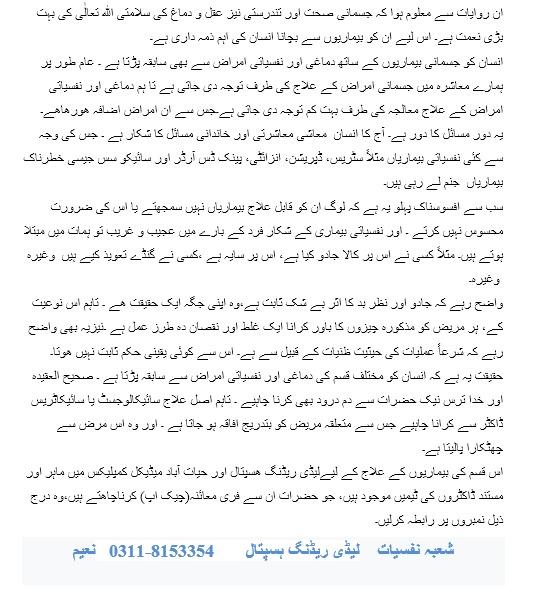


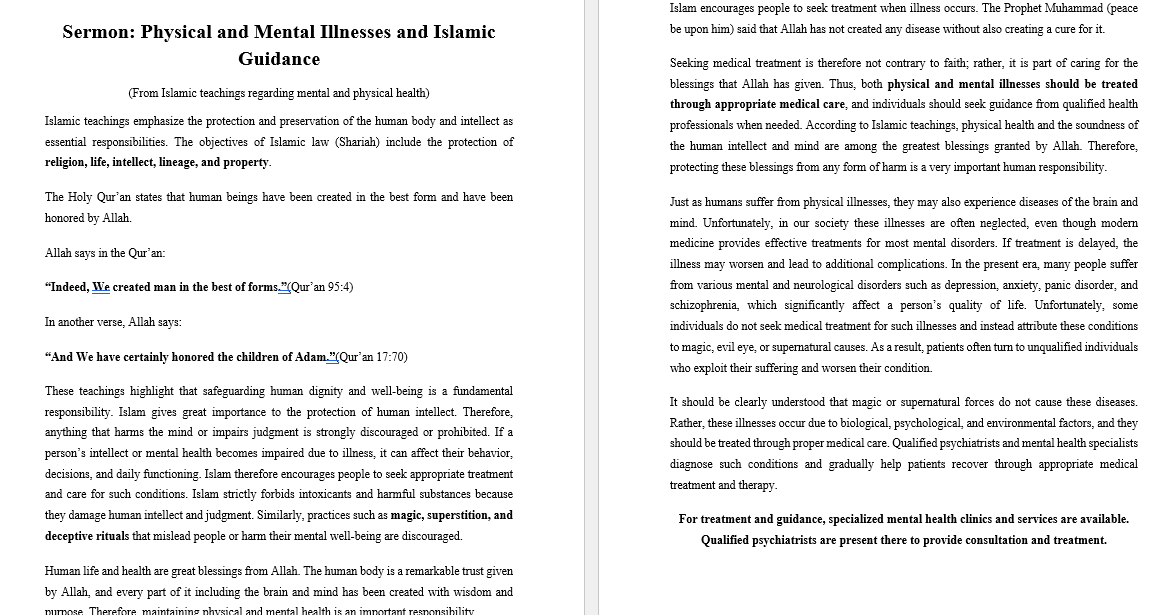


**Annex 3: Topic Guide for FGD with Parents / Carers of Psychosis Patients**

Day and Date: __________________ Time: Start ________ End __________

Moderator: _____________________ Note-Taker: _____________________

Venue: ________________________ Total Number of Participants: _______

Demographic Information (separate record of all participants to be obtained)

• Name: ________________________________

• Age: ________________________________

• Gender: ________________________________

• Location /Address: ________________________________

• Occupation: ________________________________

• Education: ________________________________

Lived Experiences of Carers of Psychosis Patients

1. How do you live your routine life with, and take care of, a psychosis patient?

2. Being a carer of a psychosis patient, what are some of the problems you have to face in day-to-day life?

Symptoms and Behavior of Psychosis Patient

3. Would you please describe some of the common symptoms expressed by your patients?

4. What are some of the ‘triggers or situations in which the patient starts behaving abnormally?

Stigma and People Perception of Psychosis

5. Would you kindly share how the patient was first diagnosed and what was the reaction of family members towards the patient?

6. What is the general reaction of people in society and neighborhood regarding psychosis patient and the family?

Late Detection and Treatment of the Patient

7. Would you please share the treatment history of the patient and how he/she was ultimately brought to the hospital?

8. If you have consulted TSHs for the treatment of the patient, how would you describe your experience?

9. In your opinion, what are the causes responsible for delay in diagnosis and treatment of psychosis patients?

10. Based on your personal observations and experiences of being a carer of psychosis patient, what do you suggest for early detection and treatment of psychosis among adolescents?

TSHs Working with Primary Care Physicians

11. Do you think it is possible and desirable for Traditional and Spiritual Healers to work with doctors and psychiatrists for early detection and treatment of Psychosis?

12. Is there anything else anyone of you want to add to the discussion?

Thank you very much for your cooperation.

**Annex 4: Topic Guide for FGD with Traditional and Spiritual Healers**

Day and Date: __________________ Time: Start ________ End __________

Moderator: _____________________ Note-Taker: _____________________

Venue: ________________________ Total Number of Participants: _______

Demographic Information (separate record of all participants to be obtained)

• Name: ________________________________

• Age: ________________________________

• Gender: ________________________________

• Location /Address: ________________________________

• Occupation: ________________________________

• Education: ________________________________

Perception about Spiritual disorders / Spirit Possession among Adolescents

1. What are the most common types of mental Illnesses or ‘possession’ experience by adolescent patients between 14 and 25 years of age?

2. What the demographic characteristics of people suffering from spirit possession?

(age, gender, economic status, education, rural-urban, etc.)

3. What are the most common symptoms and signs of spirit possessions (or Jinn – supernatural being)?

4. Why do you think adolescents are possessed by Jinn – supernatural being?

Techniques and Methods used by TSH for Treatment of Spiritual/mental Illnesses

5. Would you kindly share some of the common techniques and methods you use for treatment of such patients?

6. How effective these treatment process and methods are?

7. Are there any side-effects of the treatment you provide to patients?

Attitude of TSH towards Psychosis and Psychiatry

8. How do you view the Mental illness and modern psychiatry?

9. How often do you come across patients who you think should consult a psychiatrist? Do you refer such cases to a psychiatrist?

Willingness to work with PCP and Psychiatrists

10. If given a chance, would you be willing to work in collaboration with doctors and psychiatrists?

11. Would you be willing to get some training from doctors and psychiatrists on mental illness? On what conditions, if any?

12. Before we close this discussion, is there another thing you want to share?

Thank you very much for your cooperation.

**Annex 5: Topic Guide for FGD with Psychiatrists**

Date: __________________ Time: Start ________ End __________

Moderator: _____________________ Note-Taker: _____________________

Venue: ________________________ Total Number of Participants: _______

Demographic Information (separate record of all participants to be obtained)

• Name: ________________________________

• Age: ________________________________

• Gender: ________________________________

• Location /Address: ________________________________

# **Perception about ‘Spirit Possession’ and TSH**

1. Would you please share your personal perception about spirit possession and do deal with patients possessed by Jinn – supernatural being (if any)?

2. What do you think about Traditional and Spiritual Healers and their methods of treating patients with mental disorders?

3. Why do you think patients of psychosis usually seek treatment from traditional and Spiritual Healers (TSHs)?

# **Stigma and Suggestions for Minimizing It**

4. To what extent is mental illness a stigma in our society and how it effects the patient and their family?

5. The Duration of Untreated Psychosis (DUP) is much longer in poor countries like Pakistan as compared to developed countries. In your opinion, what could be the possible reasons for late detection and treatment of psychosis in our country?

6. What do you suggest to minimize stigma around psychosis and how to ensure early detection and treatment of the disease?

# **Psychiatrists working with PCP and TSH**

7. Would you be willing to work in collaboration with PCP and TSHs for early detection and treatment of psychosis among adolescents?

8. Can you describe any challenges or conflicts you've faced when working with traditional and spiritual healers, and how you've navigated them?

9. What, in your opinion, could be the best possible mechanism for establishing collaboration among TSHs and PCP?

10. What kind of training do you think psychiatrists should provide to PCP and TSH for early detection and referral of psychosis patients?

11. Before we close this discussion, is there another thing you want to share?

Thank you very much for your cooperation.
